# Diversity and Biocontrol Potential of Fungi Associated with Cyst Nematodes and Soils in Swiss Potato Agroecosystems

**DOI:** 10.3390/plants14243775

**Published:** 2025-12-11

**Authors:** Andrea Caroline Ruthes, Paul Dahlin

**Affiliations:** 1 Mycology, Plant Protection, Agroscope, Müller-Thurgau-Strasse 29, 8820 Wädenswil, Switzerland; 2 Entomology and Nematology, Plant Protection, Agroscope, Müller-Thurgau-Strasse 29, 8820 Wädenswil, Switzerland; paul.dahlin@agroscope.admin.ch

**Keywords:** biological control, cyst nematodes, fungal antagonists, sustainable nematode management, agroecological strategies

## Abstract

Cyst nematodes are persistent soilborne pests that severely impact crop productivity worldwide. Their protective cysts enable long-term survival and host diverse fungal communities that remain largely unexplored as potential sources of biological control agents. In this study, we isolated culturable fungi from cysts of *Globodera*, *Heterodera*, and *Punctodera*, as well as from soils collected across Swiss potato fields between 2018 and 2024. Sequencing identified 78 fungal operational taxonomic units (OTUs), predominantly belonging to Ascomycota (73%), mainly Sordariomycetes (59%) and Eurotiomycetes (8%), with additional representatives from Mortierellomycota and Basidiomycota. *Fusarium* was the most abundant genus, followed by *Clonostachys*, *Chaetomium*, and *Pochonia*, while 28% of isolates remained unclassified, indicating potentially novel taxa. Selected fungi, including *Orbilia brochopaga* CH-02, *Clonostachys rosea* CH-04 and CH-15, and *Pochonia chlamydosporia* CH-51, significantly reduced motility, infection and root galling of *Meloidogyne incognita* in vitro and in planta. Notably, CH-02 reduced root galling by 63%, highlighting its strong mechanical and antagonistic activity. These results demonstrate that cyst nematodes harbor a rich and functionally diverse fungal community with substantial biocontrol potential, providing a foundation for developing sustainable and environmentally friendly alternatives to chemical nematicides in crop protection.

## 1. Introduction

Cyst nematodes (*Globodera*, *Heterodera* and *Punctodera*) rank among the most specialized and destructive plant-parasitic nematodes, occurring in temperate [1], tropical and subtropical regions [2]. They cause substantial yield losses in crops of major economic importance, ranging from 10% to as high as 80%, affecting potatoes [3], soybeans [4,5], and other crops such as carrots, beets, brassicas, and cereals [6,7]. The long-term persistence in soil is largely attributed to the protective cyst and from robust dormancy systems, including genetically programmed diapause and environmentally induced quiescence, which together prevent hatching until appropriate seasonal and host-derived cues are present, thereby allowing eggs to survive for many years in the absence of a host and making population eradication extremely challenging, once populations are established [6,7,8,9,10,11,12]. This persistence highlights the need for sustainable, long-term management strategies.

Current control strategies combine crop rotation, resistant cultivars, chemical nematicides, and biological control agents. However, each of these approaches faces increasing limitations. Stricter pesticide regulations, climate change-driven shifts in nematode dynamics, and the emergence of resistance-breaking pathotypes, nematode populations capable of overcoming plant resistance genes that are normally effective against standard populations, further reduce the reliability of existing tools. While rising soil temperatures may accelerate development in some nematode species, several cyst nematodes, including *Globodera rostochiensis* and *Globodera pallida*, exhibit defined optimal temperature ranges beyond which reproduction declines. In addition, *Globodera* species require a winter chilling period to break egg dormancy. Therefore, milder winters may delay or partially prevent hatching, potentially altering early-season host–parasite interactions and population dynamics [13,14]. Limited crop rotation options and low genetic diversity in resistant cultivars further exacerbate the problem, while the development of new resistant cultivars remains slow and costly [15]. Collectively, these factors emphasize the urgent need for ecologically grounded strategies within integrated pest management.

Cysts provide a unique ecological niche for fungi, as their long-term persistence allows the accumulation of diverse microbial associates, some of which may have evolved antagonistic traits by exploiting nematode eggs as a resource [16]. While several cyst-associated fungi exhibit nematophagous or antagonistic activity, many isolates are primarily saprophytes or opportunistic colonizers rather than obligate parasites. Their presence on cysts may reflect ecological opportunism, exploiting available organic resources, rather than a strict parasitic lifestyle. Nonetheless, repeated recovery of certain taxa suggests potential functional relevance for nematode suppression under specific environmental conditions. Previous studies have shown that cyst-associated fungi are taxonomically diverse, including genera such as *Clonostachys*, *Cylindrocarpon*, *Diheterospora*, *Exophiala*, *Fusarium*, *Ilyonectria*, *Mortierella*, *Nematophthora*, *Neocosmospora*, *Neonectria*, *Phoma*, *Pochonia*, *Pyrenochaeta*, *Purpureocillium*, *Sarocladium*, *Setophoma*, *Sporothrix*, and *Stagnospora*, among others [17,18,19,20,21,22,23,24,25,26,27,28]. These fungi act through a wide range of mechanisms, including nematode-trapping structures [29], endoparasitism [30], egg or female parasitism [31,32,33], and the production of nematicidal metabolites [34,35], often targeting multiple nematode life stages [36].

Despite this diversity, relatively few fungal isolates have progressed into commercial biocontrol products due to limited host specificity [37,38], variable field efficacy [38,39], poor soil establishment [40,41], incompatibility with agricultural inputs [42,43], and challenges in production, formulation and shelf life [44,45,46]. Regulatory requirements and the need for performance consistency further slow adoption [47,48].

At Agroscope, the Nematology group processes 3500 to 4000 soil samples per year, primarily from seed and staple potato fields. These samples originate from all major seed potato-growing regions of Switzerland, encompassing distinct climatic zones (cool–humid alpine foothills, temperate midlands, and warmer lowland areas), a broad range of soil types (including sandy, loamy, and clay-rich agricultural soils), and multiple years of sampling (2018–2024). This geographic, pedological, and temporal breadth supports the generalizability of the observed fungal diversity patterns while still allowing the detection of site-specific associations when present.

In recent years, we focused efforts on cysts showing visible fungal colonization and expanded our analysis beyond *Globodera* to include *Heterodera* and *Punctodera* and their associated soils. By combining isolation and diversity profiling with functional testing, this study aimed to identify fungal isolates with potential biocontrol activity and to evaluate their antagonistic effects against *Meloidogyne incognita*. Although the fungal isolates were obtained from cyst nematodes (*Globodera*, *Heterodera*, and *Punctodera*), testing against *M. incognita* provides a model to assess cross-species activity, i.e., the ability of these fungi to suppress nematodes beyond their original hosts. This approach links mechanistic evaluation with broader agroecosystem relevance and provides insights into the potential applicability of cyst-associated fungi across multiple nematode pests. This approach allows evaluation of both mechanical and biochemical modes of antagonism in a well-characterized nematode system, linking mechanistic insights to broader agroecosystem relevance and potential application across multiple cropping systems.

This work provides a systematic bioprospective analysis of cyst-associated fungi, characterizing their taxonomic diversity and functional potential, and testing selected isolates for nematode suppression. The study offers insights into the ecological roles of these fungi in temperate agroecosystems and identifies promising candidates for integration into sustainable nematode management strategies.

## 2. Results

### 2.1. Fungal Diversity Associated with Cyst Nematodes and Soil Samples

**Figure 1:**
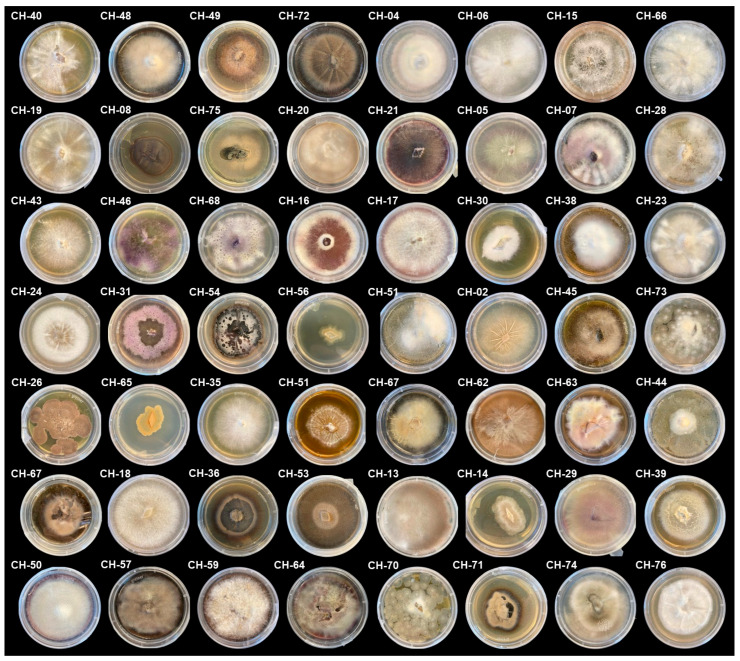
Morphological diversity of fungal isolates obtained from cyst nematodes and soil samples collected in potato-producing regions of Switzerland. Representative morphotypes are shown; for isolate details see Supplementary Table S1.

A total of 78 fungal operational taxonomic units (OTUs) were isolated and sequenced from potato field soils and cyst nematodes collected between 2018 and 2024. Of these, 50 originated from soil samples, 21 from *Globodera* cysts, 4 from *Heterodera* cysts, and 3 from *Punctodera* cysts (Supplementary Table S1). The morphological diversity of isolates is shown in Figure 1.

**Figure 2:**
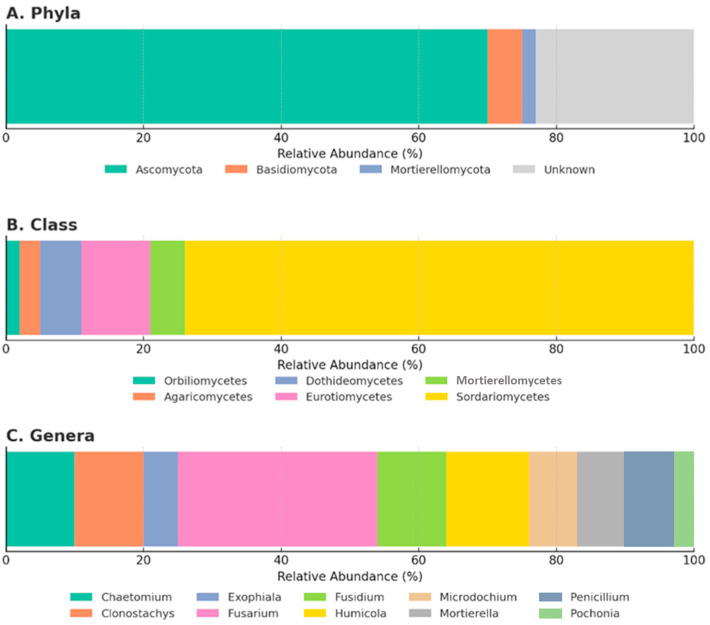
Taxonomic composition of fungal operational taxonomic units (OTUs) isolated from cyst nematodes and associated soils. Distribution is shown at the level of (**A**) phylum, (**B**) class, and (**C**) most frequent genera. The category “Unknown” includes OTUs with <97% Web BLAST identity or unresolved genus-level assignment (see Supplementary Table S1).

Most isolates (73%) belonged to phylum Ascomycota, followed by the phylum Mortierellomycota (4%) and Basidiomycota (3%) (Figure 2A). The remaining 21% could not be confidently classified due to low sequence identity (<97%) or ambiguous genus-level resolution. Within the phylum Ascomycota, the class Sordariomycetes dominated (59%), with additional representatives of the classes Eurotiomycetes (8%), Dothideomycetes (5%), and Orbiliomycetes (1%). Agaricomycetes represented the phylum Basidiomycota, while Mortierellomycetes represented the phylum Mortierellomycota (Figure 2B).

The most frequently recovered genus in Sordariomycetes was *Fusarium* (15%), followed by *Humicola*, *Chaetomium*, *Clonostachys*, *Fusidium*, and *Pochonia* (Figure 2C). The class Eurotiomycetes included *Exophiala*, *Penicillium*, and *Marquandomyces*; Dothideomycetes included *Pleospora*, *Setophoma*, and *Phaeospheria*. *Arthrobotrys* was the only Orbiliomycetes recovered. The class Agaricomycetes included *Bjerkandera* and *Trametes*, while the class Mortierellomycetes were solely represented by *Mortierella*. Overall, 28% of isolates remained unclassified (Supplementary Table S1). It should be noted that the genera listed here represent only a small fraction of the overall diversity within their respective classes and phyla.

*Fusarium culmorum* and *Trametes versicolor* were recovered from *Punctodera* cysts, *Exophiala equina* from *Heterodera*, and a diverse set of fungi, including *Clonostachys rosea*, *Dactylonectria* sp., and various *Fusarium* spp., from *Globodera* cysts (Supplementary Table S1).

### 2.2. Functional Insights and Biocontrol Potential

**Figure 3:**
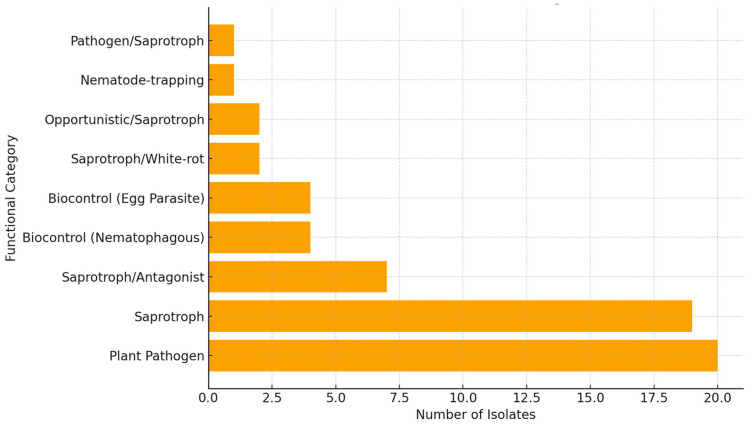
Functional distribution of fungal isolates associated with cyst nematodes in Switzerland. Bar plot showing the number of fungal isolates assigned to each functional category, based on literature-derived ecological trait mapping. Functional groups include Nematophagous fungi, Plant Pathogens, Saprotrophs/Decomposers, Endophytes, and Unknown/Unassigned taxa. Functional roles were inferred using genus- and species-level ecological information from peer-reviewed studies and curated fungal trait databases. The figure summarizes the dominant ecological guilds represented in the isolate collection and highlights taxa with potential relevance for nematode biocontrol.

Functional annotation revealed that most taxa belonged to plant-pathogenic species, such as *Fusarium* (*F. culmorum*, *F. oxysporum*, *F. poae*, *F. solani*), *Setophoma terrestris*, *Microdochium*, and *Thelonectria* (Figure 3; Supplementary Table S1). Several isolates belonged to genera containing saprotrophic groups, including *Mortierella*, *Humicola*, *Lasiosphaeria*, *Podospora*, *Trametes*, and *Penicillium*. A subset of isolates corresponded to fungal groups with reported antagonistic or nematophagous traits, including *Clonostachys rosea*, *Marquandomyces marquandii*, *Orbilia brochopaga*, and *Pochonia chlamydosporia*.

Based on this functional annotation, three isolates representing distinct ecological categories, *Clonostachys rosea*, *Orbilia brochopaga*, and *Pochonia chlamydosporia*, were selected for in vitro and in planta experimental evaluation.

### 2.3. In Vitro Evaluation of Fungal Isolates Against Meloidogyne incognita

**Figure 4:**
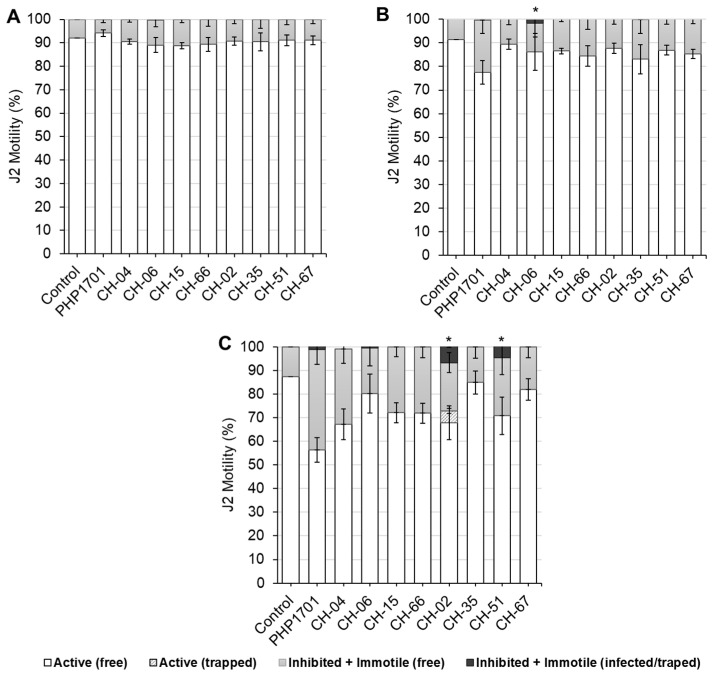
In vitro nematicidal activity of selected fungal isolates against *Meloidogyne incognita* juveniles after 1 (**A**), 3 (**B**), and 6 (**C**) days of exposure. The negative control lacked fungal treatment, while PHP1701 (*Clonostachys rosea*) served as the positive control. Error bars represent standard deviations (n = 6). Asterisks indicate statistically significant differences relative to the control (one-way ANOVA, Tukey’s HSD, *p* < 0.05).

The motility of *M. incognita* J2 was assessed at 1, 3, and 6 days post-incubation with selected fungal isolates (Figure 4A–C; Supplementary Table S2). On day 1, minimal effects were observed, with more than 88% of J2 remaining active across treatments (Figure 4A).

**Figure 5:**
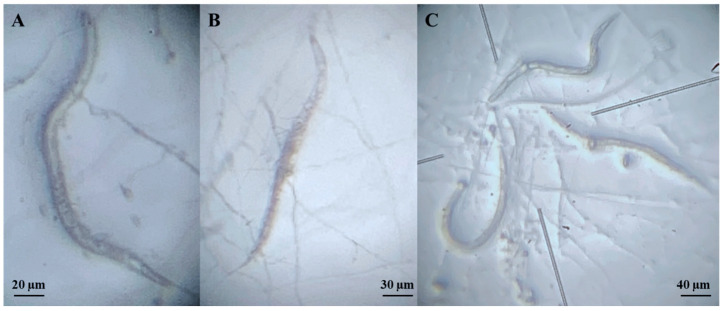
Functional interactions of fungal isolates with *Meloidogyne incognita* second-stage juveniles (J2). (**A**) *Clonostachys rosea* CH-06 showing hyphal attachment to the J2 cuticle, indicating early stages of parasitism. (**B**) *C. rosea* PHP1701 (positive control) demonstrating infection structures penetrating J2, consistent with its known parasitic activity. (**C**) *Orbilia brochopaga* CH-02 forming characteristic constricting rings around J2, providing direct evidence of mechanical trapping. Observations were made under an inverted light microscope (Zeiss, 10× magnification).

By day 3, moderate reductions in J2 motility were observed for some isolates (CH-06, CH-15, CH-66, and CH-35), while the positive control PHP1701 and isolate CH-35 caused significant reductions relative to the untreated control (Figure 4B). Visible infection was observed for CH-06 (Figure 5A).

By day 6, strong suppression of J2 motility was observed for PHP1701 and isolates CH-04 and CH-02 (Figure 4C). Visible infection or physical entrapment was confirmed for PHP1701 (Figure 5B), CH-06, CH-02, and CH-35. Notably, CH-02 (*Orbilia brochopaga*) formed mechanical trapping structures (Figure 5C).

*C. rosea* isolates showed variable effects, the positive control PHP1701 induced strong early suppression, whereas CH-04 and CH-06 showed more moderate effects. Among *P. chlamydosporia* isolates, CH-51 caused the most significant reduction in J2 motility, while CH-35 and CH-67 had limited effects.

**Figure 6:**
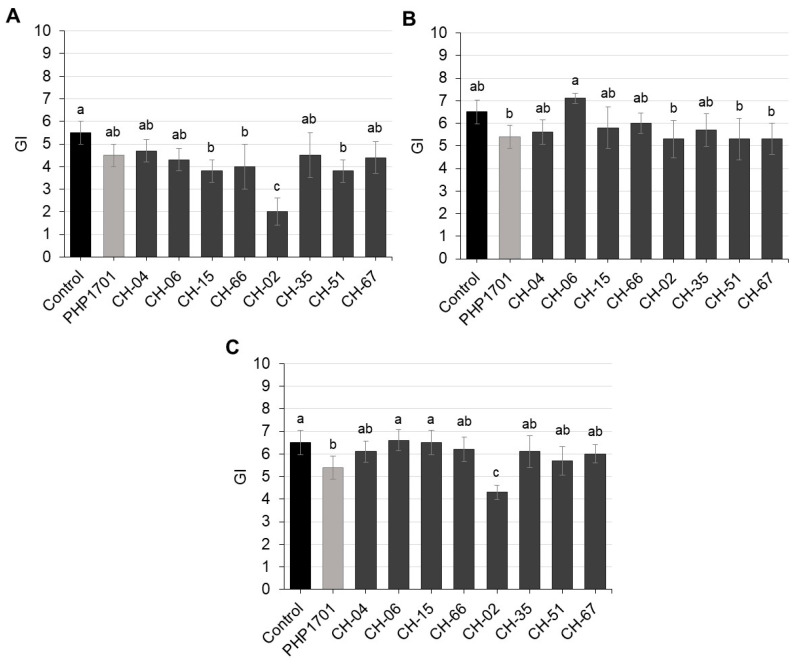
Gall index (GI) of cucumber seedlings 21 days after inoculation with *M. incognita*. Treatments included (**A**) in vitro pre-exposure of J2, (**B**) in planta inoculation with eggs (n = 5), and (**C**) in planta inoculation with J2 (n = 5). The negative control lacked fungal treatment; PHP1701 (*C. rosea*) served as the positive control. Error bars represent standard deviations. Different letters denote statistically significant differences among treatments (one-way ANOVA, Tukey’s HSD, *p* < 0.05).

Following the 6-day incubation, J2 from each treatment were transferred onto pre-germinated cucumber seedlings, and root galling was evaluated 21 days later (Figure 6A; Supplementary Table S3). The untreated control reached a high gall index (GI = 5.5 ± 0.5). Several fungal treatments reduced gall formation (Figure 6A; Supplementary Table S3).

### 2.4. In Planta Assessment of the Biocontrol Potential of Fungal Isolates Against Meloidogyne incognita

CH-02 showed the strongest effect, reducing the GI to 2.0 ± 0.6, followed by PHP1701, CH-15 and CH-66 (Supplementary Figure S1). *P. chlamydosporia* CH-51 reduced galling to GI = 3.8 ± 0.5, whereas CH-35 and CH-67 had negligible effects.

Following the in vitro screening, the selected isolates were tested in planta against *M. incognita* eggs and J2 on cucumber. Nematode infectivity varied significantly among treatments and was influenced by both the fungal isolate and the nematode life stage (Figure 6B,C; Supplementary Figures S2 and S3; Supplementary Table S3). Untreated controls reached high GI (6.5 ± 0.5), while the positive control PHP1701 reduced root galling moderately (GI 5.4 ± 0.5). CH-02 was particularly effective against J2 (GI = 4.3 ± 0.5; egg inoculation GI = 5.3 ± 0.5). *C. rosea* isolates CH-04 and CH-15 reduced galling moderately (GI: 5.6 ± 0.7 and 5.8 ± 1.1), whereas CH-06 and CH-66 were ineffective (GI ≥ 6.6). *P. chlamydosporia* isolates reduced galling to some extent (GI range: 5.3–6.1), with stronger effects in egg-inoculated treatments.

**Figure 7:**
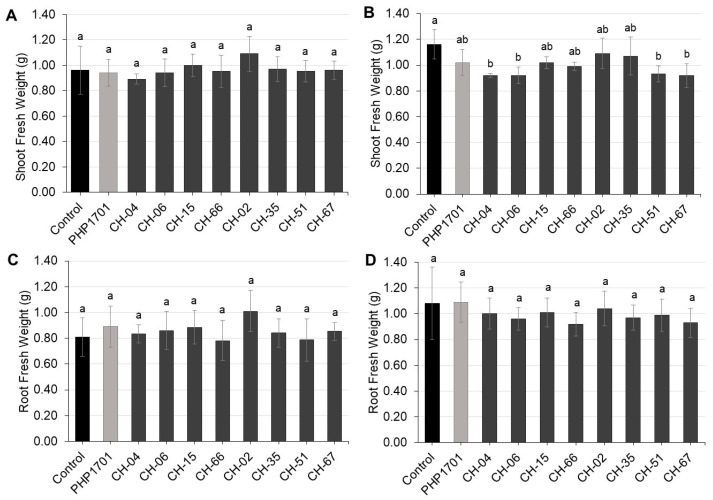
Shoot (**A**,**C**) and root (**B**,**D**) fresh weight of cucumber plants from in planta assays inoculated with *M. incognita* eggs (**A**,**B**) or J2 (**C**,**D**) (n = 5). The negative control lacked fungal treatment; PHP1701 (*C. rosea*) served as the positive control. Error bars represent standard deviations. Different letters denote statistically significant differences among treatments (one-way ANOVA, Tukey’s HSD, *p* < 0.05).

Shoot and root fresh weights (0.89 to 1.09 g) did not differ significantly from those of the untreated controls (Figure 7; Supplementary Table S4).

## 3. Discussion

### 3.1. Fungal Diversity Associated with Cyst Nematodes and Soil Samples

The fungal communities associated with cyst nematodes and potato field soils in this study were dominated by Ascomycota, particularly Sordariomycetes with additional contributions from Mortierellomycota and Basidiomycota. This composition aligns with previous surveys of nematode-associated fungi in agricultural soils, which consistently report Ascomycota dominance and frequent occurrence of genera such as *Fusarium*, *Clonostachys*, and *Chaetomium* [28,49]. The recovery of plant-pathogenic taxa, including *Fusarium* spp. and *Setophoma terrestris*, is consistent with prior findings in potato agroecosystems, while the presence of saprotrophic fungi, such as *Mortierella* and *Trametes*, reflects opportunistic colonization of cysts reported in earlier studies [50,51].

A substantial fraction of isolates remained unclassified, highlighting the largely unexplored diversity of fungi specifically associated with cyst nematodes in Swiss potato fields. Many sequences correspond to generic uncultured fungi, reflecting limited ITS representation in public databases and the possibility of previously undescribed taxa. This observation aligns with metagenomic studies reporting largely unexplored fungal diversity in nematode-associated microhabitats [52] and underscores the potential to discover novel taxa with antagonistic traits that could contribute to sustainable nematode management. Integration of high-throughput sequencing with functional assays could further uncover novel antagonists suitable for biocontrol applications.

Functional annotation identified taxa with known nematophagous or antagonistic potential, including *Clonostachys rosea*, *Pochonia chlamydosporia*, and *Orbilia brochopaga*, consistent with prior reports of these genera as biological control agents against plant-parasitic nematodes [53,54,55,56,57,58]. Although research on fungi associated with *Punctodera* cysts remains limited, likely due to its narrow host range, restricted geographic distribution, and comparatively low pathogenicity relative to *Heterodera* and *Globodera*. Consequently, most previous studies have focused on *Heterodera* and *Globodera* mycobiomes. While many isolates in this study are known as cosmopolitan saprophytes with widespread distribution [59,60,61], their repeated recovery suggests ecologically relevant associations rather than incidental colonization, potentially reflecting evolutionary selection for traits that favor antagonism toward cyst nematodes [44,62]. Several isolated fungi are already recognized as established biocontrol agents or producers of nematicidal metabolites.

Among the isolates, *Clonostachys rosea* is well-known for its broad antagonistic potential, combining direct parasitism with the production of nematicidal metabolites. Studies using in vitro assays, greenhouse experiments, and soil or root systems have documented antagonistic effects against several plant-parasitic nematode genera, including *Meloidogyne*, *Heterodera* and *Pratylenchus*, although the targeted nematode species, experimental conditions, and measured endpoints differ among these studies [53,54,63,64,65,66,67,68], and the breadth of host range inferred here reflects the combined evidence across these different experimental approaches rather than a single comparative trial. Efficacy was often strain-dependent, highlighting the importance of intra-species variation in infection dynamics and metabolite profiles. *P. chlamydosporia*, a specialized egg parasite, was highly effective against eggs of *Globodera* [68], *Heterodera* [19,69,70,71,72,73,74], and *Meloidogyne* [75,76,77], and its activity may also involve rhizosphere colonization and indirect plant-mediated defense mechanisms. *O. brochopaga*, a nematode-trapping fungus, employs constricting-ring traps to immobilize motile juveniles, particularly *Meloidogyne* and *Heterodera* J2, illustrating the effectiveness of mechanical predation as a biocontrol strategy [78,79]. Other isolated fungi, including *Fusarium*, *Exophiala*, *Mortierella*, *Penicillium*, and *Trametes* species, displayed potential antagonistic activity through mechanisms such as egg parasitism [27,31,80,81] metabolite production [82,83,84,85], or indirect plant-beneficial effects [81,86,87] but their functional roles are less well characterized.

Overall, these findings demonstrate that potato field soils and cyst nematodes harbor a rich and high potential functionally diverse fungal community. The recovery of both well-established biocontrol fungi and lesser-known taxa underscores the ecological consistency of these associations across nematode hosts and regions. The coexistence of pathogenic, mutualistic, and nematophagous fungi highlights their multifunctional roles in shaping soil and plant health and emphasizes the promise of these communities as reservoirs of novel agents for sustainable nematode management.

### 3.2. In Vitro Evaluation of Fungal Isolates Against Meloidogyne incognita

In vitro assays revealed a temporal pattern of nematode suppression, with minimal effects at day 1, moderate reductions by day 3, and strong suppression by day 6. This mirrors observations in other nematophagous fungi, which require time for conidial germination, hyphal development, and host engagement [37,88]. The mechanical trapping behavior of *O*. *brochopaga* (CH-02), including constriction and immobilization of J2, is consistent with classical predatory mechanisms reported for Orbiliomycetes [88]. The superior reduction in J2 motility by CH-02 highlights its potential as a physical biocontrol agent.

*C. rosea* strains displayed marked intraspecific variability, consistent with previous reports that strain-specific differences in metabolite production and rhizosphere competence strongly influence nematode antagonism [53,54]. Early infection by CH-06 may reflect enhanced hyphal penetration or secretion of extracellular hydrolases and secondary metabolites [89], although these mechanisms were not directly quantified here. *P. chlamydosporia* CH-51 reduced J2 motility, suggesting broader antagonistic effects beyond its specialized egg parasitism, potentially through direct parasitism or indirect plant-mediated defense responses [56,90].

The discrepancy between in vitro inhibition and in planta protection observed for some *C. rosea* isolates underscores the complexity of biological control efficacy, which depends not only on virulence but also on rhizosphere competence, persistence, and plant–microbe–nematode interactions [53,54,67]. Overall, the integration of in vitro and in planta assays provided complementary insights into fungal biocontrol potential, emphasizing the importance of mechanistic understanding and ecological compatibility when selecting strains for nematode management.

The reduction in nematode motility, infection, and root galling observed for several fungal isolates suggests antagonistic activity against *Meloidogyne incognita*. While the precise mechanisms remain to be fully elucidated, potential factors may include production of lytic enzymes, secondary metabolites, or direct parasitism of nematode juveniles. Future studies integrating enzyme assays, metabolite profiling, and microscopy will be necessary to confirm the specific modes of action underlying these biocontrol effects.

### 3.3. In Planta Assessment of the Biocontrol Potential of Fungal Isolates Against Meloidogyne incognita

Collectively, our results expand the understanding of cyst nematode-associated fungal diversity in Swiss potato fields and highlight the potential of *O. brochopaga* as a promising biocontrol agent, complementing well-established antagonists such as *C. rosea* and *P. chlamydosporia*.

In planta evaluations confirmed that CH-02 significantly reduced root galling, outperforming some *C. rosea* and *P. chlamydosporia* isolates, while shoot and root biomass remained unaffected. These findings are consistent with prior studies reporting moderate gall suppression by nematophagous fungi and highlight the importance of isolate-specific variability in efficacy [53,54,66,67].

Variation in gall suppression among fungal isolates highlights the influence of fungal species, strain, and nematode developmental stage on biocontrol efficacy. CH-02 was particularly effective against J2, consistent with its mechanical predation strategy, while *C. rosea* isolates showed strain-specific variability in gall suppression, consistent with differences in rhizosphere competence, endophytic colonization, and expression of hydrolytic enzymes or polyketide-derived metabolites [52,90,91,92]. *P. chlamydosporia* isolates were more effective against egg inoculations, aligning with their known specialization as egg parasites [56,93], and their potential to contribute to long-term reduction in egg banks across cropping cycles [94], highlighting their value in integrated nematode management strategies.

Stage-specific responses indicate that motile juveniles are generally more susceptible to physical trapping, enzymatic degradation, or interference with chemotactic signaling [95,96]. None of the fungal treatments negatively affected plant biomass, confirming the absence of phytotoxic effects. Although the fungal isolates in this study were obtained from cyst nematodes. Their antagonistic activity was evaluated against *M. incognita*, due to its global relevance, and well-characterized infection dynamics. These assays provide important insights into cross-species activity, but direct efficacy against the original cyst nematode hosts remains to be determined. Future studies will extend in vitro and in planta testing to these cyst nematodes to confirm host-specific biocontrol potential and to explore the ecological breadth of these fungal isolates.

Overall, CH-02 emerged as a particularly promising candidate against J2, while *P. chlamydosporia* isolates appear better suited for egg-stage suppression. *C. rosea* CH-04, with broad but moderate activity across both stages, represents a robust candidate for integration into sustainable nematode management programs. Combining fungal isolates with complementary mechanisms may enhance nematode suppression, mitigate resistance risks, and provide durable, environmentally sound alternatives to chemical nematicides [38,41].

## 4. Materials and Methods

### 4.1. Sample Processing

Air-dried soil samples (200 mL) obtained from nematode diagnostic samples from Swiss farms were processed with a MEKU automated soil sample extractor (modified Seinhorst-can; MEKU Erich Pollaehne GmbH, Wennigsen am Deister, Germany; www.meku-pollaehne.de), following the European and Mediterranean Plant Protection Organization (EPPO) Bulletin PM 7/119 protocol [97]. Between 2018 and 2024, cyst nematodes, including *Globodera rostochiensis*, *Globodera pallida*, *Heterodera* spp. and *Punctodera* spp. were manually collected and subsequently used for fungal isolation. Additionally, soil cores were collected from five locations in accordance with EPPO protocol PM9/26 (1) [98], with regulatory approval, from fields intended for seed potato production under regulation due to the detection of PCN.

Cysts were barcoded (where DNA yield allowed) to confirm host taxa (*G. rostochiensis*, *G. pallida*, *Heterodera* spp., *Punctodera* spp.), linking fungal isolates to their nematode origin. This approach allowed us to characterize cyst-associated fungi across different nematode hosts and soil types, providing a comprehensive view of their ecological distribution.

### 4.2. Fungal Isolation and Culture Preparation

Fungal isolates were obtained either directly from cyst nematodes or from soil using standard culture-dependent approaches. Surface sterilization of cysts was performed in 0.5% NaOCl for 5 min, rinsed five times in sterile distilled water, and transferred to Difco^TM^ potato dextrose agar (PDA; Becton, Dickinson and Company, Le Pont de Claix, France); 100 × 15 mm plates). PDA was supplemented with antibiotics to suppress bacterial growth: ampicillin (100 µg mL^−1^) and erythromycin (50 µg mL^−1^). Antibiotics were filter-sterilized and added to cooled (~50 °C) autoclaved PDA prior to plate pouring. Plates were incubated at 24 °C in the dark and monitored every other day; emerging colonies were subcultured to obtain axenic isolates and maintained on PDA at 24 °C.

For soil samples, serial dilution plating [99] was used to isolate culturable fungi. Each isolate was assigned a unique culture collection code. This systematic approach enabled the recovery of both dominant and rare fungal taxa from soils and cysts, highlighting fungal diversity in nematode-affected agroecosystems.

### 4.3. Nematode Meloidogyne incognita and Molecular Confirmation

A *Meloidogyne incognita* (Mi-virulent) culture was maintained on three-week-old tomato plants (*Solanum lycopersicum* cv. Oskar) in the greenhouse under 25 °C/19 °C (day/night), 60% relative humidity, and a 15/9 h light/dark cycle [100]. Eggs were extracted using 1% NaOCl [63] and second-stage-juveniles (J2) collected using a mist chamber. Nematode densities were determined using a counting chamber.

Periodic DNA barcoding confirmed the identity of *M. incognita* cultures [101], ensuring that bioassays tested the correct nematode species. Using a globally relevant root-knot nematode provided a practical model to assess cross-species fungal antagonism, linking lab-based findings to agricultural relevance.

### 4.4. In Vitro Evaluation of the Biological Control Potential of Selected Fungal Isolates Against Meloidogyne incognita

The biological control of selected fungal isolates was evaluated in vitro against *M. incognita* J2. Well-characterized nematophagous fungi, including *Clonostachys rosea* (CH-04, CH-06, CH-15, CH-66), *Orbilia brochopaga* (CH-02), and *Pochonia chlamydosporia* (CH-35, CH-51, CH-67) were tested. *C. rosea* strain PHP1701 (Andermatt Biocontrol Suisse, Grossdietwil, Switzerland [67]), was included as a positive control.

Fungi were cultured on PDA, spores/conidia harvested, suspended in Milli-Q water, and quantified (Neubauer chamber) at 1 × 10^7^ conidia mL^−1^. Six-well plates containing 1% water agar were inoculated with each isolate; negative controls contained no fungus. After 6 days at 24 °C, surface-sterilized J2 (150 per well) were added. Viability was monitored at 1, 3, and 6 days, and J2 categorized as: active, trapped/infected, inhibited non-infected, or inhibited fungus-infected.

On day 6, pre-germinated cucumber seedlings (*Cucumis sativus* cv. Landgurken) were introduced, lightly covered with sterile soil, and incubated for 21 days. Gall index (GI) was scored on a 0–10 scale [102]. Each treatment included at least two independent plates with three wells each (n = 6) to ensure replication. This assay design allowed mechanistic evaluation of fungal suppression on nematode motility and infectivity, simulating rhizosphere interactions.

### 4.5. In Planta Bioassay Evaluation of the Biological Control Potential

To further evaluate the isolates, in planta bioassays were conducted. Small pots (30 cm^3^) were filled with a thin soil layer, followed by the addition of either 250 J2 or 300 eggs of *M. incognita* in 100 µL suspension together with a 1 cm^2^ plug of fungal mycelium (grown on PDA) placed on the soil surface, and covered with ~15 cm^3^ soil (n = 5). After a 7-day pre-inoculation period at 22 °C, 60% relative humidity in darkness to allow fungal-nematode interactions, a pre-germinated cucumber seedling was planted in each pot. Plants were maintained under a 16/8 h light/dark cycle at 22 °C, 60% relative humidity for 28 days.

At harvest, roots were washed, GI scored [102], and shoot/root biomass measured to assess phytotoxicity.

### 4.6. Molecular Identification of Fungal Isolates

Fungal genomic DNA was extracted from mycelia using 50 µL Kawasaki buffer [103]. The internal transcribed spacer (ITS) region of rDNA was amplified using primers ITS1f [104] and ITS4 [105]. PCR conditions were as follows: initial denaturation at 95 °C for 15 min; 28 cycles of 94 °C for 1 min, 60 °C for 1 min, 72 °C for 1 min; final extension at 72 °C for 10 min. PCR products were purified and sequenced (Microsynth AG, Balgach, Switzerland).

Sequences were queried against NCBI GenBank database for taxonomic identification. Morphogroups with <97% sequence identity were annotated as “unknown” but assigned to the best matching genus-level taxon, while sequences ≥ 97% identity were assigned to the corresponding genus. This ensured confident taxonomic assignment, critical for linking fungal traits to nematode suppression potential.

Fungal community structure and classification were determined manually based on the recovered sequences, their GenBank matches, and information from the published literature. Relative abundance of each fungal genus or phylum was calculated as the proportion of isolates obtained from cysts or soil relative to the total number of isolates. Functional guild assignments (e.g., nematophagous, saprotrophic, pathogenic) were inferred from literature reports of the respective taxa.

No specialized bioinformatics software was used, as all analyses were based on cultured isolates and manually curated taxonomic information.

### 4.7. Statistical Analysis

Experiments were analyzed using univariate one-way analysis of variance (ANOVA). No data transformations were applied, as the raw data met the assumptions of ANOVA (normality and homogeneity of variances). Post hoc comparisons were performed using Tukey’s honestly significant difference (HSD) test at a 5% significance level (α = 0.05) and 95% confidence interval. Data are presented as mean ± standard deviation (SD), and statistically significant differences relative to the control are indicated in figures and tables. R and RStudio softwares were used.

## 5. Conclusions

This study provides the first systematic analysis of fungi associated with cyst nematodes and potato soils in Switzerland, revealing cysts as a rich ecological niche harboring functionally diverse fungal communities. We identified both well-established biocontrol taxa (*Clonostachys rosea*, *Pochonia chlamydosporia*, *Orbilia brochopaga*) and previously unclassified fungi, highlighting the largely unexplored diversity of cyst-associated fungi. Future functional screening of these uncharacterized isolates could reveal novel antagonists. Selected isolates demonstrated cross-species antagonism against *Meloidogyne incognita*, confirming the functional relevance of cyst-associated fungi for nematode suppression. Strain-specific variability emphasizes the importance of careful isolate selection for effective biocontrol. Overall, these findings provide new ecological insights and practical knowledge that can guide the development of sustainable, biologically based nematode management strategies in agroecosystems.

## Data Availability

The authors confirm that all data supporting the results of this study are available in the article and its Supplementary Material.

## Supplementary Materials

The following supporting information can be downloaded at: https://www.mdpi.com/article/10.3390/plants14243775/s1. Figure S1: Root gall formation and plant development of *Cucumis sativus* inoculated with *Meloidogyne incognita* second-stage juveniles (J2) pre-treated in vitro with selected fungal isolates. Yellow arrows indicate root-knot galls. Scale bar = 1 cm. Figure S2: Root gall formation and plant development of *Cucumis sativus* in an in planta assay following inoculation with *Meloidogyne incognita* eggs and selected fungal isolates. Yellow arrows indicate root-knot galls. Scale bar = 1 cm. Figure S3: Root gall formation and plant development of *Cucumis sativus* following inoculation with *Meloidogyne incognita* second-stage juveniles (J2) and selected fungal isolates. Yellow arrows indicate root-knot galls. Scale bar = 1 cm. Table S1: Culturable fungal isolates recovered from cyst nematodes and associated soil samples collected from Swiss potato farms. Table S2: Percentage of *Meloidogyne incognita* second-stage juveniles (J2) assigned to different motility and interaction categories after in vitro exposure to selected fungal isolates. Table S3: Root gall index (GI) on *Cucumis sativus*, assessed using Zeck’s [102] scale, following inoculation with *Meloidogyne incognita* second stage juveniles (J2) or eggs treated with selected fungal isolates either in vitro or in planta. Table S4: Shoot and root fresh weights of *Cucumis sativus* following inoculation with *Meloidogyne incognita* second-stage juveniles (J2) or eggs treated with selected fungal isolates in vitro or in planta. References [106,107,108,109,110,111,112,113,114,115,116,117,118,119,120,121,122] have been cited in the supplementary section.
