# Supplementary material for: Diversity and Biocontrol Potential of Fungi Associated with Cyst Nematodes and Soils in Swiss Potato Agroecosystems
Source: Plants (Basel). 2025 Dec 11;14(24):3775. doi: 10.3390/plants14243775 (PMC12737143; doi:10.3390/plants14243775)
Supplement: Supplementary file 1 [file plants-14-03775-s001.zip › plants-3987089-supplementary.pdf]

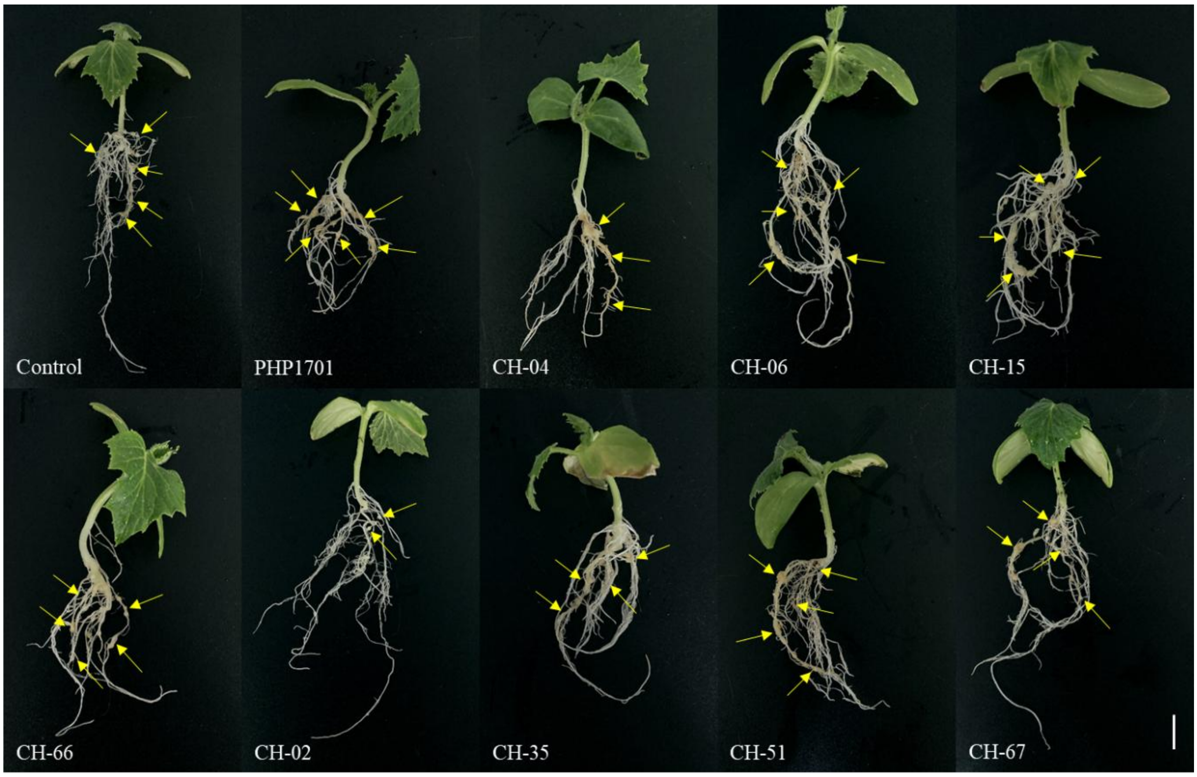

Supplementary Figure S1. Root gall formation and plant development of *Cucumis sativus* inoculated with *Meloidogyne incognita* second-stage juveniles (J2) pre-treated in vitro with selected fungal isolates. Yellow arrows indicate root-knot galls. Scale bar = 1 cm.

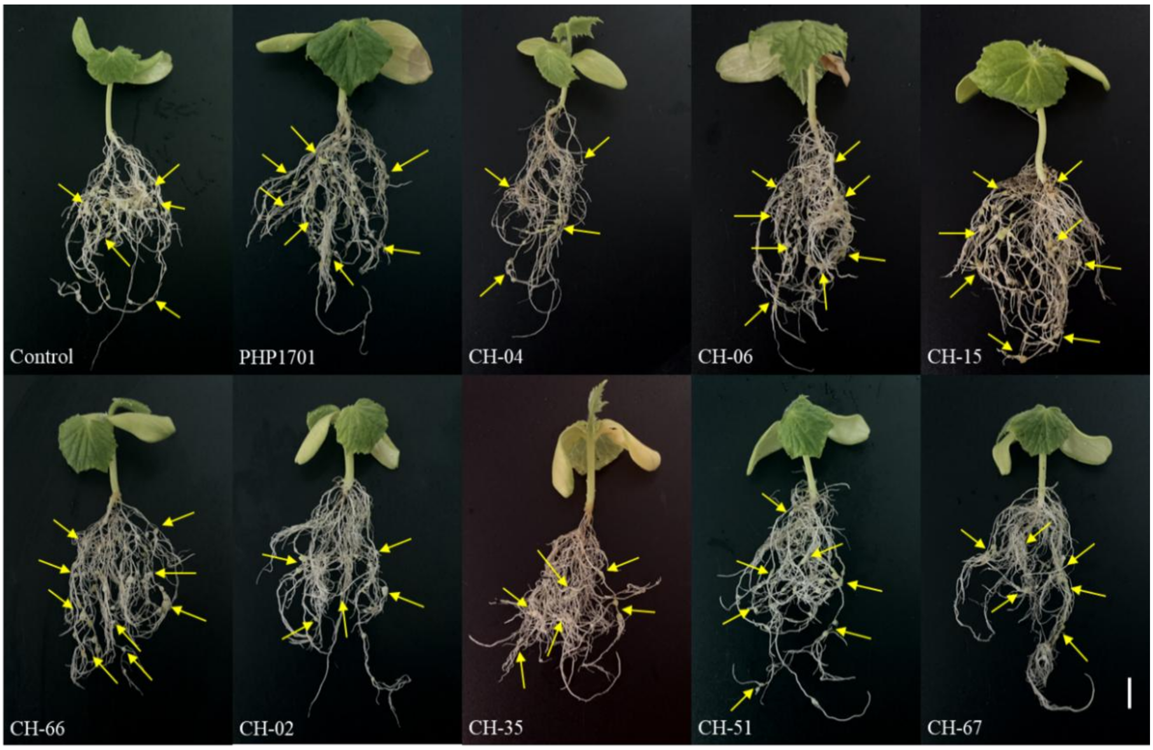

Supplementary Figure S2. Root gall formation and plant development of *Cucumis sativus* in an in planta assay following inoculation with *Meloidogyne incognita* eggs and selected fungal isolates. Yellow arrows indicate root-knot galls. Scale bar = 1 cm.

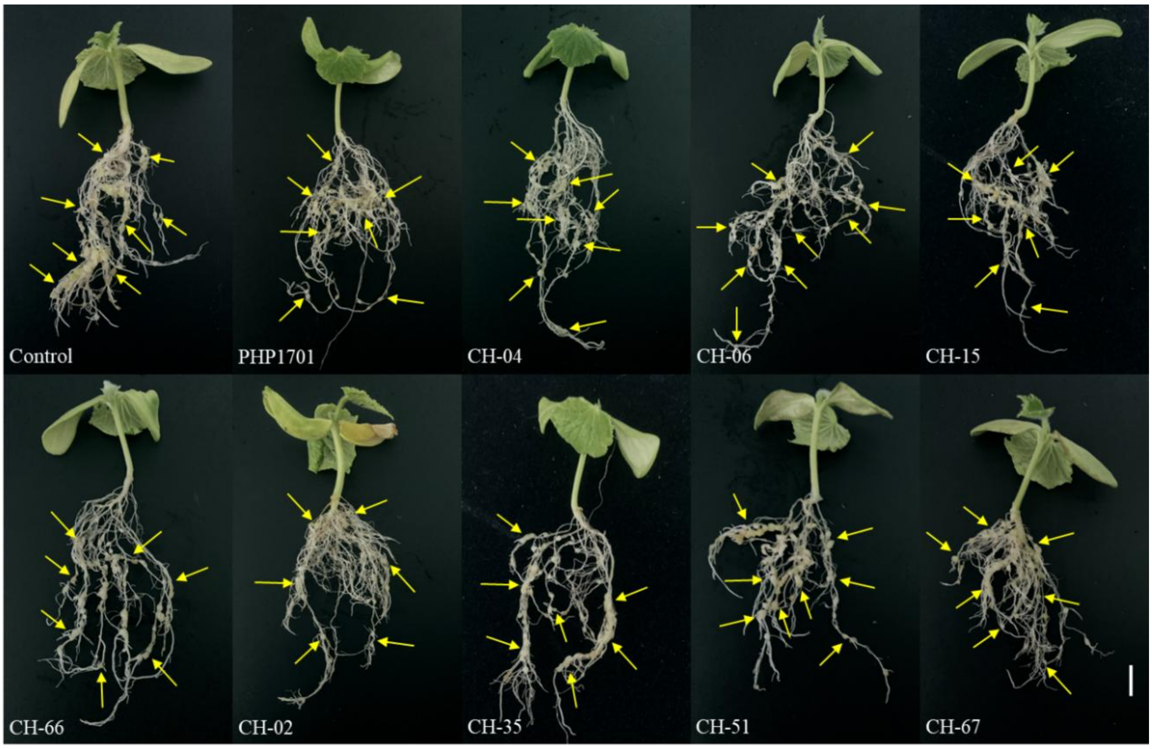

Supplementary Figure S3. Root gall formation and plant development of *Cucumis sativus* following inoculation with *Meloidogyne incognita* second-stage juveniles (J2) and selected fungal isolates. Yellow arrows indicate root-knot galls. Scale bar = 1 cm.

Supplementary Table S1. Culturable fungal isolates recovered from cyst nematodes and associated soil samples collected from Swiss potato farms.

Isolate	Operational Taxonomic Unit				Origin of fungal isolate				Functional Category	Closest Relative NCBI		Reference
	**Genus**	**Species**	**Phylum**	**Class**	***Globodera***	***Heterodera***	***Punctodera***	**Soil**		**Percent Identity**	**Accession**	
CH-01	*Bjerkandera*	*adusta*	Basidiomycota	Agaricomycetes	–	–	–	X	Saprotroph/White-rot	100.00%	OK148155.1	[52]
CH-40	*Chaetomium*	*globosum*	Ascomycota	Sordariomycetes	–	–	–	X	Saprotroph	100.00%	OR472962.1	[62,106-109]
CH-48	*Chaetomium*	sp	Ascomycota	Sordariomycetes	–	–	–	X	Saprotroph/antagonist	99.82%	PP639661.1	[29,110,111]
CH-49	*Chaetomium*	sp	Ascomycota	Sordariomycetes	–	–	–	X		100.00%	HG935478.1	
CH-72	*Chaetomium*	sp	Ascomycota	Sordariomycetes	–	–	–	X		100.00%	PP639661.1	
CH-04	*Clonostachys*	*rosea*	Ascomycota	Sordariomycetes	X	–	–	–	Biocontrol (Nematophagous)	100.00%	OM436895.1	[20,112-114]
CH-06	*Clonostachys*	*rosea*	Ascomycota	Sordariomycetes	X	–	–	–		100.00%	KR183785.1	
CH-15	*Clonostachys*	*rosea*	Ascomycota	Sordariomycetes	X	–	–	–		100.00%	MH911376.1	
CH-66	*Clonostachys*	*rosea*	Ascomycota	Sordariomycetes	–	–	–	X		100.00%	OQ816088.1	
CH-19	*Dactylonectria*	sp	Ascomycota	Sordariomycetes	X	–	–	–	Plant Pathogen	100.00%	MF440368.1	[17,29]
CH-08	*Exophiala*	*equina*	Ascomycota	Eurotiomycetes	–	X	–	–	Opportunistic/Saprotroph	100.00%	MW268795.1	[29]
CH-75	*Exophiala*	sp	Ascomycota	Eurotiomycetes	–	–	–	X	Opportunistic/Saprotroph	99.33%	KF428675.1	[115]
CH-20	*Fusarium*	*culmorum*	Ascomycota	Sordariomycetes	X	–	–	–	Plant Pathogen	100.00%	OM658376.1	[20,29]
CH-21	*Fusarium*	*culmorum*	Ascomycota	Sordariomycetes	–	–	X	–		100.00%	OM658376.1	
CH-05	*Fusarium*	*oxysporum*	Ascomycota	Sordariomycetes	X	–	–	–	Plant Pathogen	100.00%	KY765429.1	[108,110,112, 113,116-120]
CH-07	*Fusarium*	*oxysporum*	Ascomycota	Sordariomycetes	X	–	–	–		100.00%	ON411468.1	
CH-28	*Fusarium*	*oxysporum*	Ascomycota	Sordariomycetes	X	–	–	–		100.00%	MN508482.1	
CH-43	*Fusarium*	*oxysporum*	Ascomycota	Sordariomycetes	–	–	–	X		100.00%	OR478786.1	
CH-46	*Fusarium*	*oxysporum*	Ascomycota	Sordariomycetes	–	–	–	X		100.00%	OR123345.1	
CH-68	*Fusarium*	*oxysporum*	Ascomycota	Sordariomycetes	–	–	–	X		100.00%	OW986102.1	
CH-16	*Fusarium*	*poae*	Ascomycota	Sordariomycetes	X	–	–	–	Plant Pathogen	99.42%	OR562038.1	[112]
CH-17	*Fusarium*	*solani*	Ascomycota	Sordariomycetes	X	–	–	–	Plant Pathogen	100.00%	KR350652.1	[121,116,117]
CH-30	*Fusarium*	sp	Ascomycota	Sordariomycetes	X	–	–	–	Plant Pathogen	100.00%	KT313628.1	[26,29,108,117,118]
CH-78	*Fusarium*	sp	Ascomycota	Sordariomycetes	–	–	–	X		100.00%	MK407444.1	
CH-34	*Fusidium*	sp	Ascomycota	Sordariomycetes	–	–	–	X	Saprotroph	100.00%	HG936127.1	[29]
CH-38	*Fusidium*	sp	Ascomycota	Sordariomycetes	–	–	–	X		100.00%	HG936127.1	
CH-41	*Fusidium*	sp	Ascomycota	Sordariomycetes	–	–	–	X		100.00%	HG936127.1	
CH-52	*Fusidium*	sp	Ascomycota	Sordariomycetes	–	–	–	X		100.00%	HG936127.1	

Supplementary Table S1. Culturable fungal isolates recovered from cyst nematodes and associated soil samples collected from Swiss potato farms. (Continued)

Isolate	Operational Taxonomic Unit				Origin of fungal isolate				Functional Category	Closest Relative NCBI		Reference
	Genus	Species	Phylum	Class	*Globodera*	*Heterodera*	*Punctodera*	Soil		Percent Identity	Accession	
CH-11	*Humicola*	sp	Ascomycota	Sordariomycetes	X	–	–	–	Saprotroph	99.81%	OM106478.1	[116,117]
CH-12	*Humicola*	sp	Ascomycota	Sordariomycetes	X	–	–	–		99.81%	OM106478.1	
CH-23	*Humicola*	sp	Ascomycota	Sordariomycetes	X	–	–	–		99.45%	MH863772.1	
CH-24	*Humicola*	sp	Ascomycota	Sordariomycetes	X	–	–	–		99.81%	OM106478.1	
CH-27	*Humicola*	sp	Ascomycota	Sordariomycetes	X	–	–	–		100.00%	KX610414.1	
CH-33	*Lasiospheria*	sp	Ascomycota	Sordariomycetes	–	–	–	X	Saprotroph	99.19%	GU55688.1	[29]
CH-31	*Marquanomyces*	*marquandii*	Ascomycota	Eurotiomycetes	X	–	–	–	Saprotroph	99.11%	OL872352.1	[29,122]
CH-54	*Microdochium*	sp	Ascomycota	Sordariomycetes	–	–	–	X	Plant Pathogen	100.00%	AF455402.1	[29]
CH-58	*Microdochium*	sp	Ascomycota	Sordariomycetes	–	–	–	X		99.81%	GT934541.1	
CH-61	*Microdochium*	sp	Ascomycota	Sordariomycetes	–	–	–	X		99.81%	AF455402.1	
CH-56	*Mortierella*	*elongata*	Mortierellomycota	Mortierellomycotina	–	–	–	X	Saprotroph	100.00%	OQ726178.1	[115,116]
CH-42	*Mortierella*	sp	Mortierellomycota	Mortierellomycotina	–	–	–	X	Saprotroph	100.00%	MW580805.1	[26,29,109,116]
CH-47	*Mortierella*	sp	Mortierellomycota	Mortierellomycotina	–	–	–	X		99.70%	Oq726175.1	
CH-02	*Orbilia*	*brochopaga*	Ascomycota	Orbiliomycetes	–	–	–	X	Nematode-trapping	98.67%	U51950.1	[121]
CH-45	*Penicilium*	*chrysogenum*	Ascomycota	Eurotiomycetes	–	–	–	X	Saprotroph/Antagonist	100.00%	KC009774.1	[20,114]
CH-73	*Penicillium*	*chrysogenum*	Ascomycota	Eurotiomycetes	–	–	–	X		99.82%	KY218708.1	
CH-26	*Penicillium*	sp	Ascomycota	Eurotiomycetes	X	–	–	–	Saprotroph/Antagonist	99.82%	MW145187.1	[29,110,116,122]
CH-65	*Phaeospheria*	sp	Ascomycota	Dothideomycetes	–	–	–	X	Plant Pathogen	99.82%	HG936920.1	[29,115]
CH-60	*Pleospora*	sp	Ascomycota	Dothideomycetes	–	–	–	X	Pathogen/Saprotroph	100.00%	OM106317.1	[20]
CH-35	*Pochonia*	*chlamydosporia*	Ascomycota	Sordariomycetes	–	–	–	X	Biocontrol (egg parasite)	100.00%	AJ291800.1	[115]
CH-37	*Pochonia*	*chlamydosporia*	Ascomycota	Sordariomycetes	–	–	–	X		100.00%	OQ513903.1	
CH-51	*Pochonia*	*chlamydosporia*	Ascomycota	Sordariomycetes	–	–	–	X		100.00%	OQ513903.1	
CH-67	*Pochonia*	*chlamydosporia*	Ascomycota	Sordariomycetes	–	–	–	X		100.00%	OQ513903.1	
CH-62	*Podospora*	sp	Ascomycota	Sordariomycetes	–	–	–	X	Saprotroph	98.51%	PQ198855.1	[29]
CH-63	*Setophoma*	*terrestris*	Ascomycota	Dothideomycetes	–	–	–	X	Plant Pathogen	98.84%	MK012554.1	[26]
CH-44	*Thelonectria*	sp	Ascomycota	Sordariomycetes	–	–	–	X	Plant Pathogen	100.00%	MT742972.1	[29]
CH-22	*Trametes*	*versicolor*	Basidiomycota	Agaricomycetes	–	–	X	–	Saprotroph/White-rot	99.83%	KM232462.1	[29]
CH-77	*Trichocladium*	sp	Ascomycota	Sordariomycetes	–	–	–	X	Saprotroph	100.00%	MT514386.1	[29,116]

Supplementary Table S1. Culturable fungal isolates recovered from cyst nematodes and associated soil samples collected from Swiss potato farms. (Concluded)

Isolate	Operational Taxonomic Unit				Origin of fungal isolate				Functional Category	Closest Relative NCBI		Reference
	**Genus**	**Species**	**Phylum**	**Class**	***Globodera***	***Heterodera***	***Punctodera***	**Soil**		**Percent Identity**	**Accession**	
CH-18	Unknown (*Cladorrhinum*	*samala)*	Ascomycota	Sordariomycetes	X	–	–	–	Saprotroph	94.62%	FM955447.1	[29]
CH-36	Unknown (*Clohesyomyces*	sp	Ascomycota	Dothideomycetes	–	–	–	X	Saprotroph	90.35%	JQ435795.1	–
CH-53	Unknown (*Chaetomium*	sp)	Ascomycota	Sordariomycetes	–	–	–	X	Saprotroph	87.52%	KC283189.1	–
CH-13	Unknown (*Fusarium*	*oxysporum)*	Ascomycota	Sordariomycetes	–	X	–	–	Plant Pathogen	92.09%	PP213246.1	–
CH-03	Unknown	–	–	–	–	–	–	X		–	–	–
CH-09	Unknown	–	–	–	–	X	–	–		–	–	–
CH-10	Unknown	–	–	–	X	–	–	–		–	–	–
CH-14	Unknown	–	–	–	–	X	–	–		–	–	–
CH-25	Unknown	–	–	–	–	–	X	–		–	–	–
CH-29	Unknown	–	–	–	X	–	–	–		–	–	–
CH-32	Unknown	–	–	–	–	–	–	X		–	–	–
CH-39	Unknown	–	–	–	–	–	–	X		–	–	–
CH-50	Unknown	–	–	–	–	–	–	X		–	–	–
CH-55	Unknown	–	–	–	–	–	–	X		–	–	–
CH-57	Unknown	–	–	–	–	–	–	X		–	–	–
CH-59	Unknown	–	–	–	–	–	–	X		–	–	–
CH-64	Unknown	–	–	–	–	–	–	X		–	–	–
CH-69	Unknown	–	–	–	–	–	–	X		–	–	–
CH-70	Unknown	–	–	–	–	–	–	X		–	–	–
CH-71	Unknown	–	–	–	–	–	–	X		–	–	–
CH-74	Unknown	–	–	–	–	–	–	X		–	–	–
CH-76	Unknown	–	–	–	–	–	–	X		–	–	–

[1] *Bjerkandera adusta* (Willd.) P.Karst. 1879: *Boletus adustus* Willd., 1787.
[2] *Chaetomium globosum* Kunze ex Fries, 1829:
[3] *Clonostachys rosea* (Link) Schroers, Samuels, Seifert & W. Gams, 1999: *Penicillium roseum* Link, 1809*; Gliocladium roseum* Bainier. 1907, *Bionectria ochroleuca*.
[4] *Exophiala equina* (Pollacci) de Hoog, Vicente, Najafzadeh, Harrak, Badali & Seyedmousavi, 2011: *Haplographium debellae-marengoi* var. *equinum* Pollacci 1923.
[5] *Fusarium culmorum* (Wm. G. Sm.) Sacc., 1892: *Fusisporum culmorum* Wm. G. Sm., 1884.
[6] *Fusarium oxisporum* Schltdl., 1824.
[7] *Fusarium poae* (peck) Wollenw., 1913: *Sporotrichum poae* Peck, 1904.
[8] *Fusarium solani* (Mart.) Sacc., 1881: *Fusisporum solani* Mart, 1842, *Neocosmospora solani* (Mart.) L. Lombard & Crous, 2015.

[9] *Marquandomyces marquandii* (Massee) Samson, Houbraken & Luangsa-ard, 2020: *Verticillium marquandii* Masse, 1898, *Metarhizium marquandii* (Masse) Kepler, S.A. Rehner & Humber, 2014, *Paecilomyces marquandii*.
[10] *Mortierella elongata* Linnem, 1941: *Linnemannia elongata* (linnem.) Vandepol & Bonito, 2020.
[11] *Orbilia brochopaga* (Drechsler) Baral, E. Weber, Bin Liu & Z.F. Yu, 2020: *Dactylella brochopaga* Dreschsler, 1937, *Arthrobotrys brochopaga* (Drechsler) Subram., 1978, *Dreschslerella brochopaga* (Drechsler) M. Scholler, Hegedorn & A. Rubner, 1999.
[12] *Penicillium chrysogenum* Thom, 1910
[13] *Pochonia chlamydosporia* (Goddard) Zare & W. Gams, 2001: syn. *Verticillium chlamydosporium* Goddard, 1913, *Diheterospota chlamydosporia*, teleomorph *Metacordyceps chlamydosporia*.
[14] *Setophoma terrestris* (H.N. Hansen) Gruyter, Aveskamp & Verkley 2010: *Phoma terrestris* H.N. Hansen, 1929, *Pyrenochaeta terrestris*.
[15] *Trametes versicolor* (L.) Lloyd 1920: *Boletus versicolor* L., 1753, *Bjerkandera versicolor*, *Coriolus versicolor*, *Microporus versicolor*, *Poria versicolor*.

Supplementary Table S2. Percentage of *Meloidogyne incognita* second-stage juveniles (J2) assigned to different motility and interaction categories after *in vitro* exposure to selected fungal isolates.

Treatment		***M. incognita* J2 motility**			
		Active (free)	Active (trapped)	Inhibited + Immotile (free)	Inhibited + Immotile (infected/traped)
**Day 1**	Control	92.0 ± 0.0^{ab}	-	8.0 ± 0.0^{ab}	-
	PHP1701	94.1 ± 1.5^{a}	-	5.9 ± 1.5^{b}	-
	CH-04	90.5 ± 1.1^{ab}	-	9.5 ± 1.1^{ab}	-
	CH-06	89.1 ± 3.1^{b}	-	10.7 ± 2.9^{a}	-
	CH-15	88.7 ± 1.3^{b}	-	11.3 ± 1.3^{a}	-
	CH-66	89.3 ± 2.9^{b}	-	10.7 ± 2.9^{a}	-
	CH-02	90.8 ± 1.7^{ab}	-	9.2 ± 1.7^{ab}	-
	CH-35	90.4 ± 3.9^{ab}	-	9.6 ± 3.9^{ab}	-
	CH-51	91.1 ± 2.3^{ab}	-	8.9 ± 2.3^{ab}	-
	CH-67	91.1 ± 1.9^{ab}	-	8.9 ± 1.9^{ab}	-
**Day 3**	Control	91.3 ± 0.0^{a}	-	8.7 ± 0.0^{c}	-
	PHP1701	77.5 ± 5.0^{c}	-	22.1 ± 5.5^{a}	-
	CH-04	89.5 ± 2.2^{ab}	-	10.5 ± 2.2^{bc}	-
	CH-06	86.2 ± 7.8^{ab}	-	12.1 ± 5.8^{bc}	10.0 ± 0.0^{a}
	CH-15	86.5 ± 1.1^{ab}	-	13.5 ± 1.1^{bc}	-
	CH-66	84.4 ± 4.3^{abc}	-	15.6 ± 4.3^{abc}	-
	CH-02	87.7 ± 2.1^{ab}	-	12.3 ± 2.1^{bc}	-
	CH-35	83.1 ± 6.2^{bc}	-	16.8 ± 5.9^{ab}	-
	CH-51	86.9 ± 2.1^{ab}	-	13.1 ± 2.1^{bc}	-
	CH-67	85.3 ± 2.0^{ab}	-	14.7 ± 2.0^{bc}	-
**Day 6**	Control	87.3 ± 0.0^{a}	-	12.7 ± 0.0^{e}	-
	PHP1701	56.4 ± 5.3^{e}	-	42.5 ± 6.3^{a}	6.7 ± 0.0^{c}
	CH-04	67.2 ± 6.4^{de}	-	32.0 ± 6.1^{b}	-
	CH-06	80.3 ± 8.2^{abc}	-	19.3 ± 7.7^{cde}	4.0 ± 0.0^{d}
	CH-15	72.1 ± 4.2^{bcd}	-	27.9 ± 4.2^{bc}	-
	CH-66	71.9 ± 4.3^{bcd}	-	28.0 ± 4.4^{bc}	-
	CH-02	67.9 ± 7.2^{d}	5.0 ± 1.2^{a}	20.5 ± 4.1^{cde}	12.6 ± 2.3^{a}
	CH-35	84.9 ± 4.8^{a}	-	15.1 ± 4.8^{de}	-
	CH-51	70.8 ± 7.9^{cd}	-	24.7 ± 7.2^{bcd}	8.9 ± 2.8^{b}
	CH-67	82.0 ± 4.6^{ab}	-	17.9 ± 4.5^{cde}	-

Statistical differences were determined by one-way ANOVA followed by Tukey-Kramer HSD post-hoc test ($p < 0.05$, $n = 6$). Means within a column sharing the same letter are not significantly different.

Supplementary Table S3. Root gall index (GI) on *Cucumis sativus*, assessed using Zeck's (1971) scale, following inoculation with *Meloidogyne incognita* second stage juveniles (J2) or eggs treated with selected fungal isolates either *in vitro* or in planta.

Treatment	Gall Index (GI)		
	In vitro	**Eggs**	**J2**
Control	5.5 ± 0.5[a]	6.5 ± 0.5[ab]	6.5 ± 0.5[a]
PHP1701	4.5 ± 0.5[ab]	5.4 ± 0.5[b]	5.4 ± 0.5[b]
CH-04	4.7 ± 0.5[ab]	5.6 ± 0.7[ab]	6.1 ± 0.6[ab]
CH-06	4.3 ± 0.5[ab]	7.1 ± 0.9[a]	6.6 ± 0.6[a]
CH-15	3.8 ± 0.5[b]	5.8 ± 1.1[ab]	6.5 ± 0.3[a]
CH-66	4.0 ± 1.0[b]	6.0 ± 0.7[ab]	6.2 ± 0.4[ab]
CH-02	2.0 ± 0.6[c]	5.3 ± 0.5[b]	4.3 ± 0.5[c]
CH-35	4.5 ± 1.0[ab]	5.7 ± 0.9[ab]	6.1 ± 0.5[ab]
CH-51	3.8 ± 0.5b	5.3 ± 0.8[b]	5.7 ± 0.3[ab]
CH-67	4.4 ± 0.7[ab]	5.3 ± 0.4[b]	6.0 ± 0.5[ab]

Statistical significance was assessed by one-way ANOVA followed by Tukey-Kramer HSD post-hoc test ($p < 0.05$, $n = 6$). Means sharing the same letter within a column are not significantly different.

Supplementary Table S4. Shoot and root fresh weights of *Cucumis sativus* following inoculation with *Meloidogyne incognita* second-stage juveniles (J2) or eggs treated with selected fungal isolates in vitro or in planta.

Treatment	Shoot fresh weight (g)		p-value	Root fresh weight (g)		p-value
	**Eggs**	**J2**	**Egg x J2**	**Eggs**	**J2**	**Egg x J2**
Control	0.96 ± 0.19[a]	1.16 ± 0.11[a]	0.07603	0.81 ± 0.15[a]	1.08 ± 0.28[a]	0.09387
PHP1701	0.94 ± 0.11[a]	1.02 ± 0.10[ab]	0.26325	0.89 ± 0.16[a]	1.09 ± 0.16[a]	0.08351
CH-04	0.89 ± 0.04[a]	0.92 ± 0.02[b]	0.17200	0.83 ± 0.07[a]	1.00 ± 0.12[a]	0.02560*
CH-06	0.94 ± 0.11[a]	0.92 ± 0.06[b]	0.73039	0.86 ± 0.15[a]	0.96 ± 0.09[a]	0.23699
CH-15	1.00 ± 0.09[a]	1.02 ± 0.05[ab]	0.67550	0.88 ± 0.13[a]	1.01 ± 0.11[a]	0.12621
CH-66	0.95 ± 0.13[a]	0.99 ± 0.03[ab]	0.52148	0.78 ± 0.16[a]	0.92 ± 0.09[a]	0.12654
CH-02	1.09 ± 0.14[a]	1.09 ± 0.12[ab]	1.00000	1.01 ± 0.16[a]	1.04 ± 0.14[a]	0.76044
CH-35	0.97 ± 0.10[a]	1.07 ± 0.15[ab]	0.24999	0.84 ± 0.11[a]	0.97 ± 0.10[a]	0.08627
CH-51	0.95 ± 0.08[a]	0.93 ± 0.06[b]	0.66658	0.79 ± 0.16[a]	0.99 ± 0.13[a]	0.06189
CH-67	0.96 ± 0.07[a]	0.92 ± 0.09[b]	0.45537	0.85 ± 0.07[a]	0.93 ± 0.11[a]	0.04311*

Statistical significance was calculated by one-way ANOVA followed by Tukey-Kramer HSD post-hoc test ($p < 0.05$, $n = 5$). Means sharing the same letter within a column are not significantly different. In the p-value column, comparisons between Eggs and J2 treatments are shown, and an asterisk (*) indicates a significant difference when Eggs or J2 were treated with the respective fungal isolate.
